# Identification of Fitness Determinants during Energy-Limited Growth Arrest in *Pseudomonas aeruginosa*

**DOI:** 10.1128/mBio.01170-17

**Published:** 2017-11-28

**Authors:** David W. Basta, Megan Bergkessel, Dianne K. Newman

**Affiliations:** a Division of Biology and Biological Engineering, California Institute of Technology, Pasadena, California, USA; b Division of Geological and Planetary Sciences, California Institute of Technology, Pasadena, California, USA; Georgia Institute of Technology

**Keywords:** *Pseudomonas aeruginosa*, Tn-seq, fitness, growth arrest, slow growth, energy limitation

## Abstract

Microbial growth arrest can be triggered by diverse factors, one of which is energy limitation due to scarcity of electron donors or acceptors. Genes that govern fitness during energy-limited growth arrest and the extent to which they overlap between different types of energy limitation are poorly defined. In this study, we exploited the fact that *Pseudomonas aeruginosa* can remain viable over several weeks when limited for organic carbon (pyruvate) as an electron donor or oxygen as an electron acceptor. ATP values were reduced under both types of limitation, yet more severely in the absence of oxygen. Using transposon-insertion sequencing (Tn-seq), we identified fitness determinants in these two energy-limited states. Multiple genes encoding general functions like transcriptional regulation and energy generation were required for fitness during carbon or oxygen limitation, yet many specific genes, and thus specific activities, differed in their relevance between these states. For instance, the global regulator RpoS was required during both types of energy limitation, while other global regulators such as DksA and LasR were required only during carbon or oxygen limitation, respectively. Similarly, certain ribosomal and tRNA modifications were specifically required during oxygen limitation. We validated fitness defects during energy limitation using independently generated mutants of genes detected in our screen. Mutants in distinct functional categories exhibited different fitness dynamics: regulatory genes generally manifested a phenotype early, whereas genes involved in cell wall metabolism were required later. Together, these results provide a new window into how *P. aeruginosa* survives growth arrest.

## IMPORTANCE

Growth-arrested bacteria are ubiquitous in nature and disease yet understudied at the molecular level. For example, growth-arrested cells constitute a major subpopulation of mature biofilms, serving as an antibiotic-tolerant reservoir in chronic infections. Identification of the genes required for survival of growth arrest (encompassing entry, maintenance, and exit) is an important first step toward understanding the physiology of bacteria in this state. Using Tn-seq, we identified and validated genes required for fitness of *Pseudomonas aeruginosa* when energy limited for organic carbon or oxygen, which represent two common causes of growth arrest for *P. aeruginosa* in diverse habitats. This unbiased, genome-wide survey is the first to reveal essential activities for a pathogen experiencing different types of energy limitation, finding both shared and divergent activities that are relevant at different survival stages. Future efforts can now be directed toward understanding how the biomolecules responsible for these activities contribute to fitness under these conditions.

## INTRODUCTION

Microbiologists have long appreciated that most microbes on our planet spend much of their lives in a growth-arrested state due to limitation for an essential nutrient, inhibition by a toxic agent, or stalled regulatory adjustment to a new growth condition (1, 2). Importantly, cells remain viable in this state and are capable of regrowth once the limiting nutrient is replenished, the toxic inhibition relieved, or the regulatory adjustment made. Growth arrest is important in a variety of contexts, including antibiotic tolerance and persistence (3–5), establishment of mature biofilms (6–8), and ecologic biodiversity (9). For example, longer durations of antibiotic exposure select for *Escherichia coli* mutants that spend proportionally more time in growth arrest (5), *Pseudomonas aeruginosa* cells in the interior of biofilms have reduced metabolic activity and little to no growth compared to cells at the periphery (7, 8), and “seed banks” of dormant microbes contribute significantly to species richness in nutrient-poor ecosystems (10). Studies of spontaneous mutant growth during prolonged periods of starvation, referred to as the “growth advantage in stationary phase” (GASP) phenotype, have revealed how resourceful bacteria can be under periods of apparent nutrient limitation by recycling nutrients from their dying relatives (11). Yet the survival strategies that permit cells to cope when nutrients are truly scarce are poorly understood.

Microbes growing in dense communities like biofilms can quickly exhaust their electron donors or acceptors and enter growth arrest due to energy limitation. Most investigations into this state have focused on changes in cellular morphology and composition or global gene and protein expression. These studies demonstrate that energy-limited cells undergo wholesale reductions in DNA, RNA, and protein synthesis, as well as reductions in cell size and volume (12–15) and shift their regulatory landscape away from active growth to one of survival and metabolic efficiency (16). However, the functional importance of many of these structural and regulatory changes remains unclear.

While all heterotrophic microbes must contend with limiting amounts of organic carbon as an energy source, the depletion of oxygen as a terminal electron acceptor is an energy limitation specifically important for opportunistic pathogens like *P. aeruginosa*. Reduced oxygen levels have been measured in *P. aeruginosa*-colonized biofilms, cystic fibrosis sputum, and chronic wounds and likely contribute to the slow growth and antibiotic tolerance of this organism during infection (7, 17–24). In the absence of oxygen or nitrate as a terminal electron acceptor, *P. aeruginosa* enters a growth-arrested state but can survive for days to weeks if provided with pyruvate or arginine as a fermentable energy source (25, 26).

Despite the relevance of energy-limited growth arrest for *P. aeruginosa* physiology in the environment and in chronic infections, only a few studies have identified or examined genes functionally important during this state (25–31). These studies have revealed crucial metabolic pathways essential for ongoing energy generation and maintenance of the proton motive force (PMF) (25, 26) and characterized novel regulators with widespread effects on gene expression (30). However, no studies have attempted to identify genes required for fitness of *P. aeruginosa* during carbon or oxygen limitation at a genome-wide scale. A systematic investigation to identify these genes is important not only for a better understanding of basic microbial physiology, but also to determine the factors that might contribute to chronic infections caused by this organism.

In this study, we performed a functional genomic screen using transposon insertion sequencing (Tn-seq) to identify fitness determinants in *P. aeruginosa* when energy limited for organic carbon (pyruvate) as an electron donor or oxygen as an electron acceptor. Our screen reveals divergent and overlapping activities required for fitness during both types of energy limitation and highlights the value of a functional genomics approach for studying the physiology of growth arrest.

## RESULTS

### Viability and energy levels of *P. aeruginosa* during carbon or oxygen limitation.

**FIG 1:**
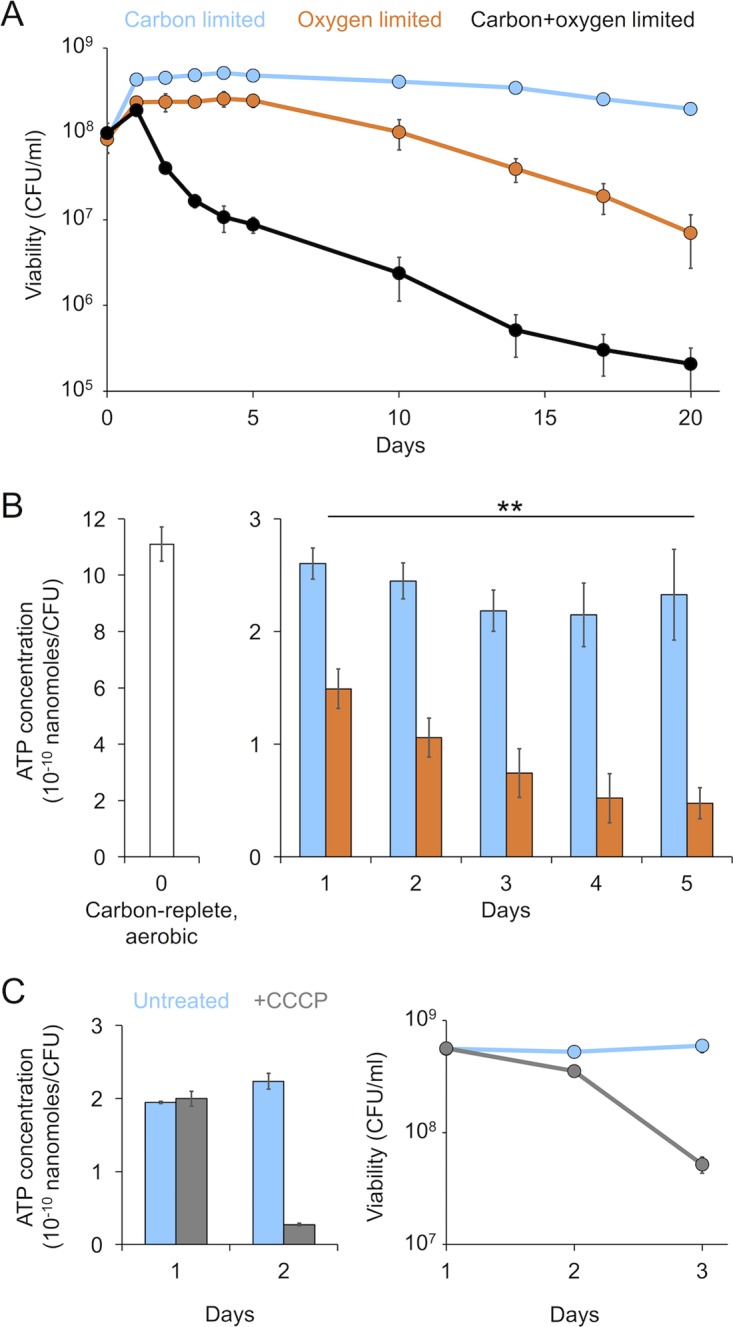
*P. aeruginosa* maintains viability at a reduced level of ATP during energy-limited growth arrest. (A) Viability of *P. aeruginosa* cultures as measured by CFU over 20 days of carbon, oxygen, or carbon and oxygen limitation. (B) Steady-state ATP concentration per CFU over the first 5 days of carbon or oxygen limitation. (C) Viability and ATP concentration of carbon-limited cells treated with 25 μM CCCP on day 1 of growth arrest. Error bars show the standard deviation of biological replicates (*n =* 3). The double asterisk indicates a significant difference in ATP concentration between carbon- and oxygen-limited cells (paired Student’s *t* test, *P* < 0.005).

We began our study by measuring the survival dynamics of *P. aeruginosa* during growth arrest caused by carbon or oxygen limitation. We chose pyruvate as the sole exogenous carbon and energy source for our experiments because *P. aeruginosa* is capable of aerobic growth as well as anaerobic survival on this substrate (25, 26). We grew cultures of the PA14 wild-type (WT) strain aerobically to the exponential phase in minimal medium with 40 mM pyruvate and then pelleted the cells and resuspended them in fresh medium with either 1 or 40 mM pyruvate. The cultures resuspended in 1 mM pyruvate were shaken aerobically for 20 days (carbon limited), while the cultures resuspended in 40 mM pyruvate were transferred into an anoxic chamber and incubated without shaking in the absence of any terminal electron acceptor for 20 days (oxygen limited). We also incubated cultures anaerobically without pyruvate to assess survival in the absence of both electron donors and acceptors (both carbon and oxygen limited). Under our experimental conditions, *P. aeruginosa* maintained viability for nearly 20 days during carbon limitation and 10 days during oxygen limitation, whereas its viability rapidly declined when limited for both carbon and oxygen (Fig. 1A). To confirm that pyruvate was completely consumed during carbon limitation, we measured its concentration using high-performance liquid chromatography (HPLC) and observed that the pyruvate concentration became undetectable by day 1 of survival, coinciding with growth arrest of the population. Addition of 40 mM pyruvate to the carbon-limited cultures on day 5 promoted outgrowth to high cell density, further confirming that pyruvate was indeed limiting for growth in our experiment (data not shown).

The difference in viability between carbon and oxygen limitation suggests that a lack of oxygen is a more severe growth-arresting trigger. We hypothesized that energy limitation might be more extreme for oxygen-limited cells because they are constrained to using the low-energy-yielding acetate kinase-phosphate acetyltransferase (AckA-Pta) pathway (25), whereas carbon-limited cells can potentially generate more energy by respiring alternative endogenous or exogenous carbon sources, such as fatty acids and amino acids derived from lipid and protein degradation, respectively (16). To test this hypothesis, we measured the steady-state ATP level during both types of energy limitation (Fig. 1B). We observed a greater than 4-fold reduction in ATP levels between actively growing (carbon-replete, aerobic) cells (day 0) and growth-arrested (carbon- or oxygen-limited) cells (days 1 to 5), confirming that carbon- or oxygen-limited cells are indeed energy limited. As predicted, ATP levels fell to an even greater extent for oxygen-limited cells by day 1 of growth arrest and continued to decline over the following days, reaching nearly 5-fold-lower levels compared to carbon-limited cells by day 5. After day 5, the viability trajectories of carbon- and oxygen-limited cells diverged, with lower viability correlated with the difference in ATP levels for these populations on day 5. To directly link ATP levels with viability, we treated carbon-limited cells with carbonyl cyanide *m*-chlorophenylhydrazone (CCCP), an ionophore that dissipates the PMF (Fig. 1C). After 1 day of CCCP treatment (day 2 of growth arrest), ATP levels were reduced 8-fold compared to those of untreated cells. This was followed by a nearly 8-fold reduction in viability on day 3. Together, these results indicate that viability is dependent on the steady-state ATP level and suggest that the reduced viability of oxygen-limited cells is due to their lower level of ATP.

### Tn-seq experimental approach.

To identify fitness determinants during carbon or oxygen limitation, we performed a genomic screen using Tn-seq. Tn-seq uses the power of massively parallel sequencing to quantify changes in relative abundance of insertion mutants in a transposon mutant library under a condition of interest (32). These changes in abundance approximate the contribution of the mutated region of DNA to growth and survival under the relevant condition. Insertion mutants with a fitness advantage increase in relative abundance, while insertion mutants with a fitness disadvantage decrease in abundance. Insertion mutants in neutral regions show no change in abundance. Tn-seq has been optimized for use in diverse bacteria and offers a high-throughput, unbiased, semiquantitative approach to interrogate the genome of an organism for fitness determinants under a variety of conditions (33).

**FIG 2:**
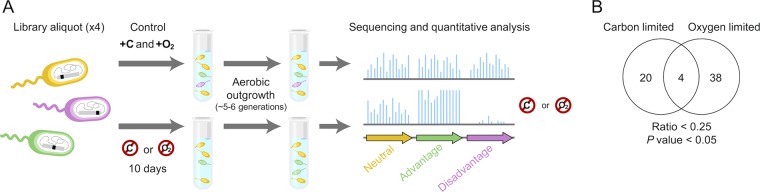
Tn-seq identifies fitness determinants during energy-limited growth arrest. (A) Diagram of the experimental approach. Each experiment was performed in duplicate. Control samples were grown only under carbon-replete, aerobic conditions, while energy-limited samples were incubated without carbon or oxygen for 10 days followed by carbon-replete, aerobic outgrowth. (B) Number of genes identified as having a greater than 4-fold significant fitness defect defined as a mean read ratio of <0.25 and a combined *P* value of <0.05.

We generated a transposon library in PA14 containing ~150,000 unique mutants using the randomly inserting Tn*5*-based transposon T8 (34). This transposon is designed such that only the gene into which it inserts is transcriptionally silenced—i.e., polar effects on downstream genes are avoided (34). We subjected replicate aliquots of our library to either carbon or oxygen limitation for 10 days and compared the reads per gene following aerobic outgrowth of these energy-limited samples to those of control samples grown only under carbon-replete, aerobic conditions (Fig. 2A [see Materials and Methods for details]). The majority of mutated genes had a mean read ratio of ~1 between the energy-limited and control samples, indicating that most insertions had a neutral effect on fitness (see Fig. S1 in the supplemental material). There were no unique insertions in any of our energy-limited samples with a read count of greater than 0.4% of the total reads in the population (where the average was ~0.0008%). Importantly, those mutants with the highest proportional read counts were present at equally high rates in all replicates of both the energy-limited and control samples. This indicates that their increased abundance was not due to an overwhelming fitness advantage caused by either the transposon insertion or a spontaneous mutation, but rather was due to an insertion “hot spot” in the initial pooled library. Together, these data strongly suggest that no significant population turnover occurred throughout the duration of our experiments due to GASP mutations.

10.1128/mBio.01170-17.1 FIG S1  General Tn-seq results. Correlation of total reads per gene between the replicates for control and carbon-limited samples (A) and control and oxygen-limited samples (B). Reads are displayed in reads per kilobase per million mapped reads (RPKM). Each point represents a single gene. For display on a logarithmic scale, genes with zero reads were given a value of 1 before RPKM conversion. (C) Frequency of read ratios between the energy-limited samples and the corresponding controls for each gene. The geometric mean of the read ratios for each replicate is plotted. (D) Cumulative number of unique insertions in our PA14 transposon library. A linear pattern indicates an even distribution of insertions across the genome. Download FIG S1, TIF file, 1.1 MB. Copyright © 2017 Basta et al. 2017 Basta et al. This content is distributed under the terms of the Creative Commons Attribution 4.0 International license.

We defined a gene as being required for fitness during carbon or oxygen limitation if the mean read ratio of the replicates was <0.25 and the combined *P* value was <0.05. This represents mutants with a greater than 4-fold fitness defect following aerobic outgrowth after 10 days of carbon or oxygen limitation and encompasses genes required for entry, maintenance, and reemergence from growth arrest. We picked these stringent criteria to limit the number of potential hits and increase the likelihood of identifying genes that would exhibit a strong phenotype; Data Sets S1 and S2 in the supplemental material show read ratios and *P* values for genes and intergenic regions across the entire genome. Based on our criteria, we identified a total of 62 required genes. Of these genes, 20 were required specifically during carbon limitation, 38 specifically during oxygen limitation, and 4 during both types of energy limitation (Fig. 2B). We did not identify any mutants with a fitness advantage during carbon limitation based on a mean read ratio of >4 and a combined *P* value of <0.05. However, 25 mutants were identified as having a fitness advantage during oxygen limitation based on these criteria (see Fig. S2 in the supplemental material).

10.1128/mBio.01170-17.2 FIG S2  Mutants with a fitness advantage during oxygen-limited growth arrest. (A) Functional categories of mutants identified as having a greater than 4-fold significant fitness advantage, defined as a mean read ratio of >4 and a combined *P* value of <0.05. (B) Fitness of the WT relative to two mutants at days 10, 20, and 30 of oxygen limitation. The double colon indicates a transposon mutant. Download FIG S2, TIF file, 1.4 MB. Copyright © 2017 Basta et al. 2017 Basta et al. This content is distributed under the terms of the Creative Commons Attribution 4.0 International license.

10.1128/mBio.01170-17.4 DATA SET S1  Read ratios for each gene and intergenic region in the carbon-limited Tn-seq experiment. Download DATA SET S1, XLSX file, 0.8 MB. Copyright © 2017 Basta et al. 2017 Basta et al. This content is distributed under the terms of the Creative Commons Attribution 4.0 International license.

10.1128/mBio.01170-17.5 DATA SET S2  Read ratios for each gene and intergenic region in the oxygen-limited Tn-seq experiment. Download DATA SET S2, XLSX file, 0.8 MB. Copyright © 2017 Basta et al. 2017 Basta et al. This content is distributed under the terms of the Creative Commons Attribution 4.0 International license.

### Identification of known fitness determinants during carbon or oxygen limitation.

**FIG 3:**
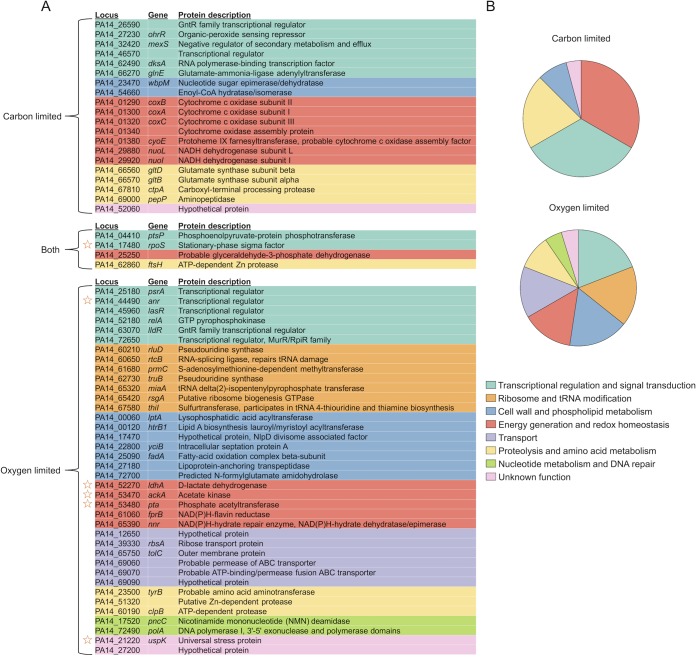
Functional categories of genes required during energy-limited growth arrest. (A) Locus, gene name, and protein description of required genes. “Both” represents genes required during carbon or oxygen limitation. Stars indicate genes that were previously described as important for fitness during energy-limited growth arrest in *P. aeruginosa*. (B) Pie chart representing the genes listed in panel A.

We identified *rpoS* as required for fitness during carbon or oxygen limitation, as expected (Fig. 3A). This conserved stress response regulator is activated upon entry into stationary phase and known to directly or indirectly regulate hundreds of genes in *P. aeruginosa* (35, 36). The functional importance of RpoS during carbon limitation has been demonstrated previously for *P. aeruginosa* using glucose as the limiting carbon source (27, 28). Finding that RpoS is also required under our experimental conditions is not surprising and thus provides an important validation of our approach.

We also identified most of the loci previously shown to have a survival defect during oxygen limitation. These include the *ackA-pta* operon, encoding acetate kinase-phosphate acetyltransferase, *ldhA*, encoding d-lactate dehydrogenase, *anr*, encoding an anaerobic transcriptional regulator, and *uspK*, encoding a universal stress protein (25, 26, 29) (Fig. 3A). We did not detect a fitness defect for insertions in the integration host factor encoded by *ihfA* and members of the *arcDABC* operon required for arginine fermentation, although both loci were previously shown to have an anaerobic survival defect on pyruvate (25, 29). However, in our screen *ihfA* appeared to be essential for growth of PA14 on pyruvate, showing few reads in the aerobically grown controls compared to the initial pooled library (see Materials and Methods and Data Sets S3 and S4 in the supplemental material). This precluded a determination of conditional fitness because there were too few reads to compare between the energy-limited and control samples. Additionally, a strain with deletion of the entire *arcDABC* operon was used in previous survival experiments (29). Because Tn-seq only assesses the fitness of single-gene mutants, this could explain why we did not detect a fitness defect in any of the *arc* genes individually. Overall, we conclude that our experimental approach is sensitive to detect individual, conditionally required genes during carbon or oxygen limitation.

10.1128/mBio.01170-17.6 DATA SET S3  Raw read counts for each gene and intergenic region in the carbon-limited Tn-seq experiment. Download DATA SET S3, XLSX file, 2.4 MB. Copyright © 2017 Basta et al. 2017 Basta et al. This content is distributed under the terms of the Creative Commons Attribution 4.0 International license.

10.1128/mBio.01170-17.7 DATA SET S4  Raw read counts for each gene and intergenic region in the oxygen-limited Tn-seq experiment. Download DATA SET S4, XLSX file, 2.4 MB. Copyright © 2017 Basta et al. 2017 Basta et al. This content is distributed under the terms of the Creative Commons Attribution 4.0 International license.

### Functional classification of fitness determinants.

We grouped genes required for fitness into eight categories to get a better sense of the activities required during energy-limited growth arrest (Fig. 3A and B). Genes were assigned to the different categories based on previous functional characterization in *P. aeruginosa* or similar annotation to a characterized gene in *E. coli*. We found that many categories were common to both types of energy limitation, whereas the specific genes in each category mostly differed. For example, among genes required for “energy generation and redox homeostasis” only *PA14_25250*, encoding a putative glyceraldehyde-3-phosphate dehydrogenase (GAPDH), was required during carbon or oxygen limitation. The *coxBAC* gene cluster, encoding subunits of the low-affinity *aa*_3_-type cytochrome *c* oxidase, and *nuoL* and *nuoI*, encoding subunits of the proton-pumping NADH dehydrogenase I (NDH-1), were specifically required during carbon limitation. Other members of the *nuo* operon had milder defects during carbon limitation that did not meet our fitness criteria (Data Set S1). On the other hand, the *ackA-pta* and *ldhA* loci, the NAD(P)H oxidoreductase encoded by *fprB*, and the NAD(P)H-hydrate dehydratase/epimerase encoded by *nnr* were all specifically required during oxygen limitation. Other categories with this pattern included “transcriptional regulation and signal transduction,” “cell wall and phospholipid metabolism,” “proteolysis and amino acid metabolism,” and “unknown function.” Notably, in the “transcriptional regulation and signal transduction” category, *dksA* was specifically required during carbon limitation, the global regulators *psrA*, *anr*, *lasR*, and *relA* were specifically required during oxygen limitation, and *rpoS* and *ptsP* were required during both types of energy limitation.

Unexpectedly, we found that the categories of “ribosome and tRNA modification,” “transport,” and “nucleotide metabolism and DNA repair” were specifically required during oxygen limitation. Among the first category were genes encoding the pseudouridine synthases RluD and TruB, the thiouridine synthase ThiI, and the isopentenyltransferase MiaA. The second category included a homologue of *E. coli tolC*, encoding an outer membrane efflux protein, and an operon encoding a putative ABC transporter (*PA14_69060*, -*69070*, and -*69090*). The third category comprised *pncC*, encoding a nicotinamide mononucleotide deamidase, and *polA*, encoding DNA polymerase I.

### Experimental validation of Tn-seq results.

**FIG 4:**
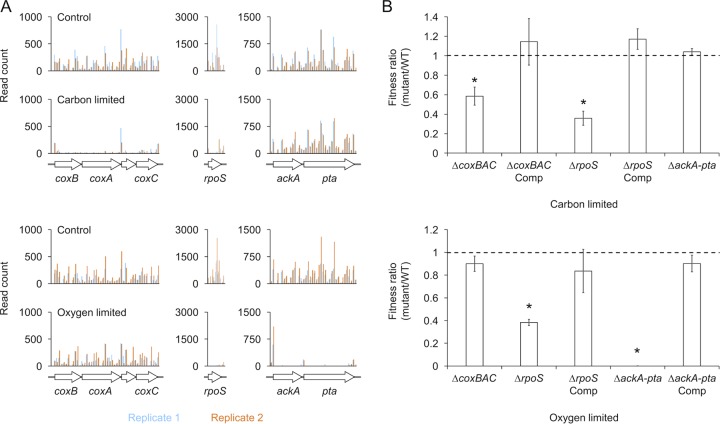
Validation of fitness determinants identified by Tn-seq. (A) Read counts for insertions across the *coxBAC*, *ackA-pta*, and *rpoS* loci required during carbon limitation, oxygen limitation, or both, respectively. Each locus is divided into 100-bp windows, and the cumulative number of insertions in each window is plotted. (B) Fitness of the corresponding deletion mutants and the complemented strains relative to the WT after 13 days of carbon limitation or 10 days of oxygen limitation. Error bars show the standard deviation of biological replicates (*n =* 3). The asterisk indicates a significant fitness defect relative to the WT (paired Student’s *t* test, *P* < 0.05).

To validate our Tn-seq results, we made strains with unmarked deletions of the *coxBAC* (including the open reading frame [ORF] *PA14_01310*), *ackA-pta*, and *rpoS* loci, required during carbon limitation, oxygen limitation, or both, respectively (Fig. 4A), and cocultured them with a fluorescently labeled WT strain to simulate the competitive environment in our screen. As predicted, a fitness defect was observed for the Δ*coxBAC* mutant specifically during carbon limitation, the Δ*ackA-pta* mutant specifically during oxygen limitation, and the Δ*rpoS* mutant during carbon or oxygen limitation (Fig. 4B). The fitness defect for each strain was rescued by complementing the deleted locus at the *att*Tn*7* site on the chromosome. These results indicate that our experimental approach is both robust and specific in its detection of genes required for fitness during energy-limited growth arrest.

### Fitness dynamics of mutants during oxygen limitation.

**FIG 5:**
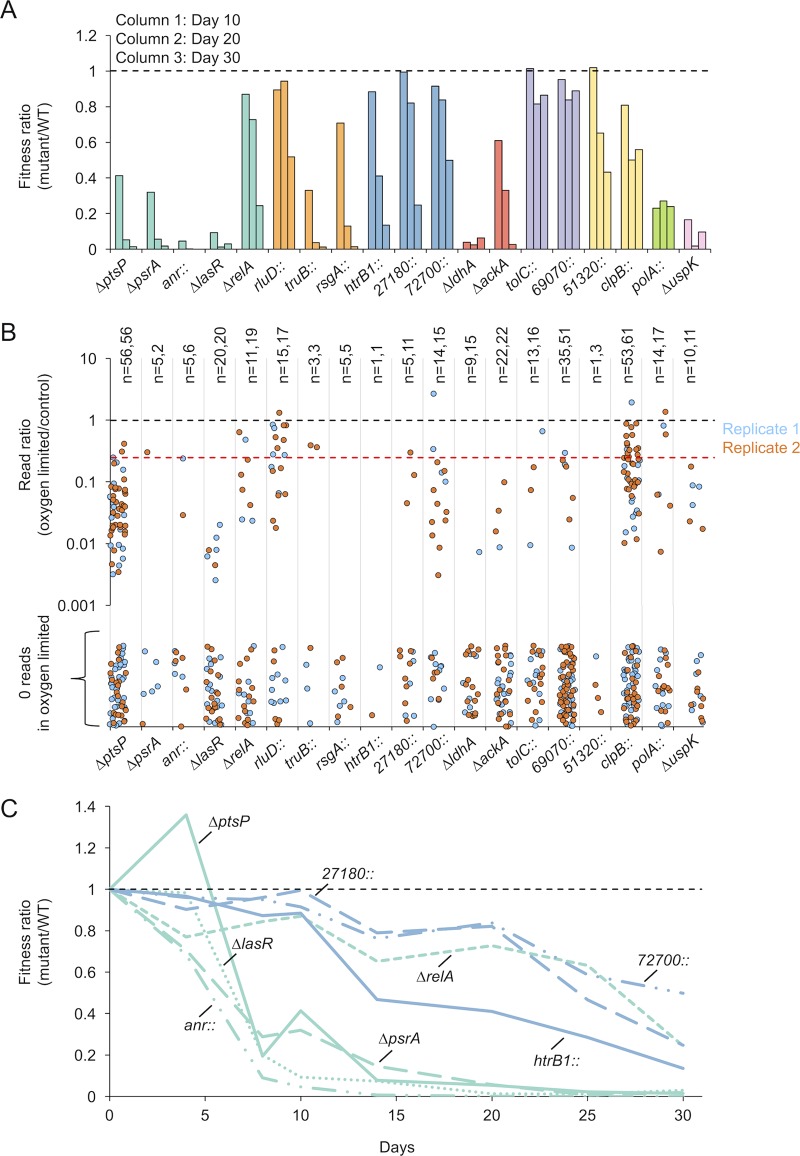
Competition between select mutants and the WT during oxygen limitation. (A) Fitness of mutants relative to the WT at days 10, 20, and 30 of oxygen limitation. Colors correspond to the functional categories described in Fig. 3. A double colon indicates a transposon mutant. (B) Read ratio of insertions in the oxygen-limited Tn-seq samples relative to the control for each mutant tested in panel A. Each point represents a unique insertion within the middle 80% of the gene. Ratios are shown for insertions with a minimum of 20 reads in the control. *n* equals the number of unique mutants in replicates 1 and 2, respectively. The dashed red line indicates a read ratio of 0.25. (C) Time course of mutant fitness relative to the WT for genes in the “transcriptional regulation and signal transduction” and the “cell wall and phospholipid metabolism” categories.

We chose to investigate genes required during oxygen limitation in more detail based on the relevance of this condition to *P. aeruginosa* physiology in biofilms and chronic infections (20–24). We retrieved transposon mutants (37) or made unmarked deletions of representative genes from each functional category required during oxygen limitation. We then competed these mutants against our fluorescent WT strain under oxygen-limited conditions and confirmed that most (17 of 19) mutants had a fitness defect; representative strains from different functional categories are shown in Fig. 5A. We noticed that some mutants had either a mild defect or no defect after 10 days of competition, but were outcompeted by days 20 and 30. In contrast, nearly all insertion mutants within each gene showed a fitness defect at day 10 in our screen (Fig. 5B). This suggests that our screen identified mutants with fitness defects earlier than might be detected by direct, one-on-one competition with the WT strain, further highlighting the sensitivity of our experimental approach.

Some mutants we tested did not have an observable fitness defect in competition with the WT strain. Of note, the two “transport” mutants showed little to no defect even at day 30 of competition (Fig. 5A, purple columns). We found this particularly surprising for *PA14_69070*, which is part of a three-member operon encoding subunits of a putative ABC transporter (*PA14_69060*, -*69070*, and -*69090*). According to our selection criteria, all three genes in this operon were required during oxygen limitation (Fig. 3A), and all mutants with independent insertions within the middle 80% of the *PA14_69070* ORF had a fitness defect (Fig. 5B). To determine if the lack of a fitness defect for *PA14_69070* in our competition experiment was due to the specific transposon mutant used, we made an unmarked deletion strain of the entire transporter operon and competed this strain against our fluorescent WT strain. To our surprise, the unmarked deletion strain also did not have a fitness defect (data not shown). This discrepancy could be a false-positive result detected in our screen or a consequence of some environmental difference between the screen and competition experiment that influences a gene’s importance. One difference is that each mutant represents a tiny fraction of the population in the screen, whereas it is closer to half the population in the competition experiment. This difference in the mutant proportion could influence community dynamics in an unforeseen way that cannot easily be captured in one-to-one competitions.

We noticed that the magnitude of the fitness defect at each time point in our competition experiment varied between the different categories (Fig. 5A). Although all of the genes we tested had a greater than 4-fold fitness defect in our screen, this observation suggested that some functional categories might be more important for fitness than others, or they might be important at different stages during oxygen limitation. To test this hypothesis, we performed the competition experiment with mutants in the “transcriptional regulation and signal transduction” and “cell wall and phospholipid metabolism” categories and took more frequent time points to monitor fitness with greater temporal resolution (Fig. 5C). As suggested by Fig. 5A, most regulatory genes were required earlier in growth arrest, being reduced to a small fraction of the population by day 10, and in the case of the *anr* mutant becoming undetectable by day 15. In contrast, genes involved in cell wall metabolism maintained nearly 100% fitness at day 10 and began to gradually decline only later in growth arrest. The exception to this general trend is the Δ*relA* mutant, which showed a milder defect compared to the other regulators. We conclude that genes belonging to multiple functional categories are required during energy-limited growth arrest and likely contribute to fitness at different stages and to various extents.

## DISCUSSION

Tn-seq is a powerful method for identifying fitness defects of individual mutants in a genetically heterogeneous population. Importantly, Tn-seq can detect these fitness defects independent of the pattern of gene expression, which is useful under stress conditions where significant posttranscriptional regulation can occur (30, 38). Previous studies have used Tn-seq to identify fitness determinants in bacteria under different growth-arresting conditions. These studies have expanded our knowledge of genes required for survival of ionizing radiation (39, 40), reactive oxygen and nitrogen species (41), antibiotics (42), and the host immune system (43, 44). To our knowledge, however, no studies have systematically identified fitness determinants during growth arrest caused by long-term energy limitation.

In this study, we used Tn-seq to identify the genes required for fitness of *P. aeruginosa* when limited for organic carbon as an electron donor or oxygen as an electron acceptor. A companion study by Pechter et al. identifies fitness determinants in *Rhodopseudomonas palustris* under carbon-limited but energy-replete conditions (45). We found that multiple general functions were important during carbon or oxygen limitation in *P. aeruginosa*, despite substantial differences in the specific genes required. Our results highlight the fundamental challenges cells face in the context of energy-limited growth arrest: maintaining the PMF and redox homeostasis, conserving ATP, efficiently repairing and synthesizing macromolecules such as proteins and DNA, and regulating these activities in a fine-tuned and concerted way. Only when these challenges are met can cells properly enter into, endure, and reemerge from growth arrest, as established for yeast over a decade ago (46).

### Two methods to maintain the PMF and ATP synthesis during energy-limited growth arrest.

The importance of continued, efficient respiration during carbon limitation is underscored by the requirement for multiple genes in the electron transport chain (ETC). The subunits of NADH dehydrogenase I (NDH-1) encoded by *nuoL* and *nuoI* and the low-affinity *aa*_3_-type cytochrome *c* oxidase encoded by the *coxBAC* gene cluster (47) were required specifically during carbon limitation. In contrast, mutations in *nuo* and the cytochrome biogenesis operon *ccm* resulted in a significant fitness advantage during oxygen limitation, suggesting that ongoing activity of the ETC is deleterious in the absence of a terminal electron acceptor (Fig. S2). Unlike NDH-2, the multisubunit NDH-1 complex catalyzes proton-coupled electron flow from NADH to ubiquinone, thereby contributing to the PMF. NDH-1 mutants of *E. coli* have a fitness defect in stationary phase (48), and the need for this complex in our study indicates that its proton-pumping activity may be crucial for survival during aerobic carbon limitation. The *coxBAC* locus is normally repressed under nutrient-replete growth conditions and induced in an RpoS-dependent manner upon starvation for carbon, nitrogen, or iron (49). The *aa*_3_ oxidase is the most efficient proton pump of the five terminal oxidases encoded by *P. aeruginosa* (50), and was recently shown to be required for fitness during carbon limitation (92). These results suggest that enhanced respiratory efficiency is essential to maintain the PMF and ongoing ATP synthesis during carbon limitation, a condition in which there is a shortage of electron donors for respiration.

In contrast to the importance of respiration during carbon limitation, pyruvate fermentation becomes an essential means of energy generation in the absence of a terminal electron acceptor. The *ackA-pta* operon, encoding acetate kinase and phosphate acetyltransferase, and *ldhA*, encoding lactate dehydrogenase, showed specific fitness defects during oxygen limitation. AckA and Pta are required for ATP generation via anaerobic pyruvate oxidation, while LdhA is required for regenerating NAD^+^ consumed in the reaction (25, 26). The ATP produced by this pathway is then used by the F_1_F_o_-ATPase to pump protons across the membrane (26). The importance of NAD^+^ regeneration is underscored by the requirement for the putative NAD(P)H oxidoreductase encoded by *fprB* and the NAD(P)H-hydrate dehydratase/epimerase encoded by *nnr*. The latter acts to repair hydrated, nonfunctional NAD(P)H using ADP as a phosphoryl donor (51). Together, these results indicate that redox homeostasis and substrate-level phosphorylation are critical for PMF maintenance and ongoing ATP synthesis during oxygen limitation, in the absence of alternative electron acceptors for respiration.

### Functional categories required specifically during oxygen limitation.

Genes involved in ribosome and tRNA modification were required during oxygen limitation but not carbon limitation (Fig. 3A and B). This could be due to the lower energy levels in oxygen-limited cells relative to carbon-limited cells (Fig. 1B). Protein synthesis is one of the most energy-intensive processes that occurs during bacterial growth and is substantially downregulated upon growth arrest (16). Ribosomal and tRNA modifications could promote the fidelity and efficiency of translation during oxygen limitation by a variety of mechanisms. In *E. coli*, for example, the methyltransferase PrmC is required for efficient translation termination (52, 53) and the pseudouridine synthase TruB functions as an essential tRNA chaperone (54, 55). These different activities could become important at the more reduced energy levels encountered during oxygen limitation, where inefficiencies in translation might have a proportionally greater impact on fitness. Another possibility is that these ribosome and tRNA modifications promote translation of specific proteins required during oxygen limitation. One example of this type of regulation is the requirement of the tRNA isopentenyltransferase MiaA for efficient RpoS expression in *E. coli* (56). Both MiaA and RpoS are required during oxygen limitation in our screen, suggesting that MiaA may function as an activator of RpoS in *P. aeruginosa* as well. Whatever the exact roles of these ribosome- and tRNA-modifying enzymes, they highlight the importance of ongoing protein synthesis during energy-limited growth arrest.

Surprisingly, genes involved in DNA repair were also specifically required during oxygen limitation. Among these genes, DNA polymerase I, encoded by *polA*, is upregulated during oxygen limitation with an excess of arginine (30). This enzyme may repair DNA damage caused by aberrant anaerobic flux through the ETC or oxidative damage upon reaeration (16). Although no genes encoding known DNA repair enzymes were required specifically during carbon limitation, we identified the putative glyceraldehyde-3-phosphate dehydrogenase (GAPDH), encoded by *PA14_25250*, as required during both types of energy limitation. GAPDH is canonically thought to be involved in central metabolism as an enzyme of glycolysis, but recent work suggests it can also moonlight as a DNA repair enzyme in *E. coli* (57). Maintaining genomic integrity is critical for survival of growth-arrested cells (16), and polymerases that respond to DNA damage are required for fitness during long-term stationary phase in *E. coli* (58). Unfortunately, Tn-seq—like any genetic screen with mutants in single loci—is not well suited to detect genes that are important but not required because other genes can play similar roles (59). This might explain why apart from GAPDH we did not detect more DNA repair enzymes as fitness determinants during carbon limitation. Alternatively, multiple nonredundant repair pathways may be operating in parallel during carbon limitation, with a mutation in any one pathway resulting in a mild or stochastic fitness defect not identifiable by our stringent selection criteria (59).

### Essentiality of proteolysis.

Multiple proteases were required during carbon or oxygen limitation, including FtsH, which was required during both types of energy limitation. Protein catabolism might be important during energy-limited growth arrest in order to remove aberrant/damaged proteins, relieve the burden of energy-intensive enzymes such as ribosomes, decrease cell mass and thus the cellular maintenance requirement, activate or deactivate regulatory proteins, or provide amino acid substrates as a biosynthetic/energy source (16, 60–63). In regard to the last function, continued protein synthesis is essential during carbon or oxygen limitation (30, 61), and the recycling of amino acids by regulated proteolysis of nonessential proteins can be an energy-efficient way to allow for ongoing synthesis of the proteins required for survival. Furthermore, recycled amino acids can be used to generate energy and may serve as one source of electron donors for continued respiration during carbon limitation (16). Consistent with this notion, *E. coli* mutants more efficient in amino acid uptake display a growth advantage in the stationary phase (64).

### A central role for RpoS.

The stationary-phase sigma-factor RpoS was required during carbon or oxygen limitation, revealing a potentially nuanced interplay between RpoS and other regulators identified in our screen (DksA, RelA, PsrA, and LasR). The RNA polymerase-binding transcription factor DksA, which we found to be specifically required during carbon limitation, acts in concert with the stringent-response modulators guanosine tetra- and pentaphosphate [(p)ppGpp] to decrease expression of rRNA in both *E. coli* and *P. aeruginosa* (65, 66) and increase expression of RpoS in *E. coli* (67, 68). This suggests that DksA might upregulate RpoS in *P. aeruginosa* as well. Regulators required during oxygen limitation with links to RpoS include the stringent-response regulator RelA, which synthesizes (p)ppGpp in response to a variety of stress conditions and is required for RpoS expression and RpoS-dependent gene regulation in *E. coli* (69, 70), the transcription factor PsrA, which directly upregulates RpoS expression in *P. aeruginosa* (71, 72), and the quorum-sensing regulator LasR. The regulon of LasR is highly interlinked with RpoS, with LasR indirectly increasing RpoS expression and RpoS regulating many quorum-controlled genes (35, 73, 74). As mentioned previously, the required tRNA modification enzyme MiaA is also important for efficient RpoS expression (56), although we did not classify it as a regulator in this study. Together, these results indicate that RpoS functions as a central regulator that interacts with distinct, condition-specific coregulators to tune downstream regulatory output.

In addition to its interplay with other regulators, RpoS might directly or indirectly regulate many genes in different categories important during energy-limited growth arrest. As mentioned above, the *coxBAC* operon is induced by RpoS upon carbon limitation (47). In addition, we found that many genes required during oxygen limitation might also be regulated by RpoS in stationary phase, based on the published data set from a recent study with PA14 (36). These genes include *thiI*, *htrB1*, *PA14_27180*, *PA14_72700*, *tolC*, *PA14_69060*, -*69070*, and -*69090*, *tyrB*, *clpB*, and *uspK*. It thus seems plausible that specific growth arrest triggers not only influence which regulatory routes lead to RpoS activation, but also which downstream genes are ultimately controlled by RpoS.

### Open questions.

An important question raised by our work is to what degree the nutritional environment influences the functions required during energy-limited growth arrest. In our experiments, we limited cells specifically for pyruvate or oxygen and showed that many different functions were required in response to each limitation. However, to what extent the genes required for fitness following limitation for pyruvate or oxygen overlap required genes following limitation for other carbon or electron-accepting sources remains an open question. For example, while RpoS is required for fitness of *P. aeruginosa* during carbon limitation for glucose or pyruvate, it is dispensable when cells are starved after growth in succinate or LB (28). Clearly, the environmental context plays an important role in the cellular response to energy limitation, and genes required for fitness will intimately depend on the experimental conditions. It therefore is not surprising that different genes exhibited different fitness dynamics in our experiments: throughout the weeks-long period of our studies, certainly the chemical milieu of our cultures was changing; such changes are known to underpin the selection of different GASP mutants in *E. coli* throughout long-term survival (75), although we do not have evidence that GASP mutants comprised a significant fraction of the population with a fitness advantage.

A complementary question that arises from our work is the generalizability of our findings to other microbes. Studies on the growth arrest physiology of the respiratory pathogen *Mycobacterium tuberculosis* have revealed results both similar to and contrasting with what we observed. Like *P. aeruginosa*, *M. tuberculosis* is capable of long-term survival during carbon or oxygen limitation (76, 77), but dies rapidly when limited for both substrates (78). Similarly, ATP levels are reduced 5- to 6-fold during these two energy-limited states compared to carbon-replete, aerobic conditions (78, 79). However, in contrast to *P. aeruginosa*, *M. tuberculosis* requires continued respiration to drive ATP synthesis during carbon or oxygen limitation, and this respiration is dependent on NDH-2 instead of NDH-1 (78, 79). Additionally, the glyoxylate shunt enzyme isocitrate lyase (ICL) is required for survival of *M. tuberculosis* during these two types of energy limitation (78, 80) but is not required in *P. aeruginosa* under the conditions of our screens. ICL is upregulated during growth arrest in *M. tuberculosis*, where it can be used to bypass steps of the tricarboxylic acid (TCA) cycle that produce reducing equivalents and can help maintain the PMF via electrogenic succinate secretion (80–82). Together, these data indicate that different microbes can exploit diverse solutions to deal with the common challenges that arise during energy limitation, such as maintaining the NAD^+^ pool and the PMF, as well as generating ATP.

### Conclusion.

Our findings contribute to a growing body of work revealing the genetic determinants of fitness during energy-limited growth arrest. Future studies will probe the molecular and biochemical bases for these fitness determinants and help us interpret why they are necessary at different stages of survival. The environmental and clinical relevance of these genes can be assessed using *in vitro* models of biofilm formation and *in vivo* models of chronic infection, complemented by *in situ* gene expression profiling in patient samples (19). For example, *rpoS* and *fadA*, both required for fitness during oxygen limitation in our screen, are upregulated in the hypoxic sputum of patients with cystic fibrosis (83, 84). Overall, our study validates Tn-seq as a powerful approach for interrogating the genome-wide fitness of *P. aeruginosa* during carbon or oxygen limitation, opening up new targets for studying how this important opportunistic pathogen survives during energy-limited growth arrest.

## MATERIALS AND METHODS

### Bacterial strains, plasmids, primers, and growth conditions.

The strains, plasmids, and primers used in this study are listed in Table S1 in the supplemental material. *E. coli* and *P. aeruginosa* were grown in lysogeny broth (LB) (Difco) or on LB agar plates at 37°C for all cloning and strain construction purposes unless otherwise noted. All growth arrest experiments were performed at 33°C. This temperature was chosen because the PA14 WT strain survived significantly better anaerobically at 33°C than at 37°C. Isolated transposon mutants retrieved from the PA14 mutant library (37) were verified by colony PCR using primers flanking the annotated insertion site. For the carbon-limited experiments, cultures were shaken at maximum speed on a standard analogue shaker (VWR).

10.1128/mBio.01170-17.3 TABLE S1  Strains, plasmids, and primers used in this study. Download TABLE S1, XLSX file, 0.1 MB. Copyright © 2017 Basta et al. 2017 Basta et al. This content is distributed under the terms of the Creative Commons Attribution 4.0 International license.

### Generation of the transposon library.

The randomly inserting Tn*5*-based transposon T8 (IS*lacZhah*-tc) was conjugated into PA14 as previously described (34). Briefly, the PA14 WT strain and *E. coli* SM10λ*pir* carrying the transposon-bearing plasmid pIT2 were resuspended in LB from overnight streak plates on LB agar or LB agar plus carbenicillin (100 μg/ml), respectively. The resuspended cultures were mixed in a 2:3 PA14/SM10λ*pir* ratio based on optical density at 500 nm (OD_500_), spot plated onto LB agar plates, and incubated 2 h at 37°C. Following incubation, spots were pooled from the plates and resuspended thoroughly in LB. The pooled resuspension was diluted to an OD_500_ of ~1.75, and aliquots were plated on LB agar plus tetracycline (60 μg/ml) and chloramphenicol (10 μg/ml) to select for transposon PA14 transposon insertion mutants. The plates were incubated 24 h at 37°C. Following incubation, colonies from all plates were pooled and resuspended in phosphate-buffered saline (PBS) plus 25% glycerol. The density of the pooled library was adjusted to an OD_500_ of 8 and stored as 1-ml aliquots at −80°C. The library consisted of ~150,000 unique mutants, as determined by the number of pooled colonies and the number of unique insertions identified by sequencing.

### Tn-seq sample preparation.

Four aliquots of the transposon library were thawed on ice for 15 min and diluted to a starting OD_500_ of 0.05 in 50 ml of minimal medium (26) supplemented with 40 mM sodium pyruvate (Sigma) as the sole carbon and energy source. The cultures were grown aerobically at 37°C for ~2 generations to an OD_500_ of 0.2. Cells were pelleted and resuspended in 50 ml of minimal medium supplemented with either 40 mM pyruvate for the oxygen-limited samples or 1 mM pyruvate for the carbon-limited samples (two replicates for each condition). Immediately upon resuspension, an aliquot of each sample was diluted to a starting OD_500_ of ~0.00625 in 25 ml of minimal medium supplemented with 40 mM pyruvate. These diluted samples served as the control and were grown aerobically at 37°C with shaking for ~5 to 6 generations to an OD_500_ of 0.2. Following aerobic outgrowth, 10 ml of the control samples was pelleted and stored at −80°C. From the remainder of the resuspended cultures, 25 ml of the oxygen-limited samples was placed in a Balch tube and transferred into a glove chamber (Coy) containing an atmosphere of 15% CO_2_, 80% N_2_, and 5% H_2_. The tubes were stoppered and incubated anaerobically at 33°C. For the carbon-limited samples, 5 ml of the resuspended cultures was placed in a 25-ml test tube and incubated aerobically at 33°C and 50% relative humidity with shaking. On day 10 of incubation, an aliquot of each culture, both oxygen limited and carbon limited, was diluted to a starting OD_500_ of ~0.00625 in 25 ml of minimal medium supplemented with 40 mM pyruvate. These diluted samples were grown aerobically at 37°C with shaking for ~5 to 6 generations to an OD_500_ of 0.2. Following aerobic outgrowth, 10 ml of the energy-limited samples was pelleted and stored at −80°C. A sample of one thawed transposon library aliquot was also collected to determine the makeup of the initial pooled library.

### Sequencing and data analysis.

Genomic DNA was extracted from the pelleted samples using the DNeasy blood and tissue kit (Qiagen) and prepared for Illumina sequencing according to established protocols (85). Briefly, genomic DNA was sheared by sonication to produce 200- to 500-bp fragments and end repaired using the NEBNext end repair module (New England Biolabs). A poly(C) tail was added to the end-repaired DNA using a terminal deoxynucleotidyl transferase (Promega). C-tailed DNA was amplified in two rounds of PCR to enrich for transposon-genome junctions and to add adapters for Illumina sequencing. The amplified DNA was sequenced using 100-bp single-end reads on the Illumina HiSeq 2500 platform at the Millard and Muriel Jacobs Genetics and Genomics Laboratory at Caltech. Sequences were mapped to the UCBPP-PA14 genome sequence using Bowtie (86) and analyzed using the ARTIST Tn-seq analysis pipeline in MatLab (87). Briefly, total reads mapping to each gene in the carbon- or oxygen-limited samples were compared to their corresponding reads in the control samples using a Mann-Whitney U statistical test (87). After each replicate was analyzed independently, the geometric mean of the read ratio was calculated for each gene in both replicates and the *P* values were combined using Fisher’s combined probability test (Data Sets S1 and S2). Mutants that dropped out during growth on LB or pyruvate minimal medium overlapped with those previously identified as essential genes in *P. aeruginosa* (Data Sets S3 and S4) (88).

### Strain construction.

Unmarked deletions in PA14 were made as previously described (30) with minor modifications. Briefly, ~1-kb fragments immediately upstream and downstream of the target locus were amplified by PCR and joined with the suicide vector pMQ30 (89) (cut with SacI and HindIII) using Gibson assembly (90). The assembled construct was transformed into *E. coli* DH10B, and transformants were plated on LB plus gentamicin (20 μg/ml). For all plasmids, a correctly assembled construct was identified by colony PCR using primers flanking the multiple-cloning site and then verified by sequencing (Retrogen). Triparental mating was performed to conjugate the construct into the PA14 WT. Merodiploids containing the chromosomally integrated construct were then selected on Vogel-Bonner minimal medium (VBMM) plus gentamicin (80 μg/ml) (91). Merodiploids were grown to exponential phase in LB and counterselected on LB agar plates lacking NaCl and containing 10% sucrose. Deletions were identified by colony PCR using primer sets both flanking and internal to the target locus.

To complement the deletion strains, the genomic region of the deleted locus was amplified by PCR and joined with the shuttle vector pUC18T-mini-Tn*7*T (91) (cut with SacI and HindIII) using Gibson assembly. The assembled construct was transformed into *E. coli* DH10B, and transformants were plated on LB plus gentamicin (20 μg/ml). Tetraparental mating was performed to conjugate the construct into the corresponding deletion strain. Conjugants were selected on Vogel-Bonner minimal medium (VBMM) plus gentamicin (80 μg/ml), and chromosomal integration at the *att*Tn7 site was detected by colony PCR as previously described (91).

To make the WT strain constitutively expressing mApple, a 1-kb fragment upstream of the ribosomal protein-encoding gene *rpsG* and a 155-bp fragment immediately downstream were amplified by PCR and joined as flanking sequences to the amplified mApple open reading frame (ORF) (Addgene) in pUC18T-mini-Tn*7*T (cut with SacI and HindIII). The assembled construct was integrated into the *att*Tn*7* site in the same manner as described for the complementation strains.

### Viability measurements.

CFU were determined over time for the WT strain during carbon and/or oxygen limitation by taking a 20-μl aliquot of the growth-arrested cultures and performing serial dilutions in aerobic pyruvate media. Appropriate dilutions were plated as 10-μl drips on LB agar plates and incubated aerobically at 37°C. Colonies were counted after overnight incubation, and the number of CFU per milliliter was calculated.

### ATP measurements.

Measurement of ATP was performed as previously described (26). Briefly, a 20-μl aliquot of the growth-arrested cultures was added to 180 μl of dimethyl sulfoxide (DMSO). The samples were then diluted with 800 μl of 100 mM HEPES (pH 7.5) and stored at −80°C until analysis. ATP was measured by mixing thawed samples 1:1 with BacTiter-Glo reagent (Promega) in a 96-well opaque white microtiter plate. Luminescence was measured at 30°C using a plate reader (BioTek). A standard curve was generated with each plate measurement using known concentrations of ATP.

### Pyruvate measurements.

Samples were collected by centrifuging 300 μl of culture and pipetting 250 μl of the supernatant into a fresh tube. The samples were stored at −80°C until analysis. Thawed samples were mixed 1:1 with 100 mM H_2_SO_4_ and transferred to an autosampler vial. Pyruvate was measured by HPLC using a Waters Alliance e2695 separations module and 2998 photodiode array detector. Separations were performed using an Aminex HPX-87H column (Bio-Rad) with an isocratic elution of 5 mM H_2_SO_4_ at 0.5 ml/min and 30°C. The injection volume was 20 μl, and the total run time was 40 min. Compounds were detected at 206 and 322 nm. Pyruvate was identified by comparing retention times to a pure standard, as well as by a distinctive absorbance peak around 320 nm.

### Competition assays.

WT cells constitutively expressing an mApple fluorescent marker and individual markerless mutants were grown aerobically at 37°C in 3 ml of 40 mM pyruvate medium to an OD_500_ of between 0.1 and 0.4. Cells were pelleted and resuspended in minimal medium supplemented with either 40 mM sodium pyruvate for the oxygen-limited samples or 1 mM sodium pyruvate for the carbon-limited samples to achieve an OD_500_ of 0.2. After resuspension, the fluorescent WT was mixed with each of the markerless mutants in a 1:3 ratio of WT to mutant. An aliquot of each mixture was diluted to a starting OD_500_ of ~0.00625 in 2 ml of 40 mM pyruvate medium. These dilutions served as time zero for competition and were grown aerobically at 37°C with shaking for ~5 to 6 generations to an OD_500_ of 0.2. From the remainder of the mixtures, 1 ml of the oxygen-limited samples was placed in an Eppendorf tube and transferred into a glove chamber (Coy) containing an atmosphere of 95% N_2_ and 5% H_2_. The tubes were incubated anaerobically at 33°C. For the carbon-limited samples, 5 ml of the mixtures was placed in a 25-ml test tube and incubated aerobically at 33°C and 50% relative humidity with shaking. Subsequent time points were taken by diluting an aliquot of the growth-arrested cultures to a starting OD_500_ of ~0.00625 in 2 ml of 40 mM pyruvate medium. These dilutions were grown aerobically at 37°C with shaking for ~5 to 6 generations to an OD_500_ of 0.2. Following aerobic outgrowth, a small aliquot of the cultures was taken for epifluorescence microscopy using a Zeiss Axio Imager microscope. The ratio of cells with and without fluorescence in each mixed culture was determined for each time point and divided by the ratio at time zero. A markerless WT strain was also competed against the fluorescent WT strain as a control to normalize the ratios for the WT-mutant mixtures. The fluorescent WT strain had a mild fitness defect in competition with the markerless WT strain during oxygen limitation.
