# Erratum for Basta et al., “Identification of Fitness Determinants during Energy-Limited Growth Arrest in *Pseudomonas aeruginosa*”

**DOI:** 10.1128/mBio.00576-18

**Published:** 2018-04-10

**Authors:** David W. Basta, Megan Bergkessel, Dianne K. Newman

**Affiliations:** a Division of Biology and Biological Engineering, California Institute of Technology, Pasadena, California, USA; b Division of Geological and Planetary Sciences, California Institute of Technology, Pasadena, California, USA

## ERRATUM

Volume 8, no. 6, e01170-17, 2017, https://doi.org/10.1128/mBio.01170-17. The following should be added to the end of Acknowledgments: “This study was supported by NIH grants 5R01AI127850 and 5R01HL117328 to D.K.N.”

