# Supplementary figures and images for: Identification of Fitness Determinants during Energy-Limited Growth Arrest in Pseudomonas aeruginosa
Source: mBio. 2017 Nov 28;8(6):e01170-17. doi: 10.1128/mBio.01170-17 (PMC5705914; doi:10.1128/mBio.01170-17)

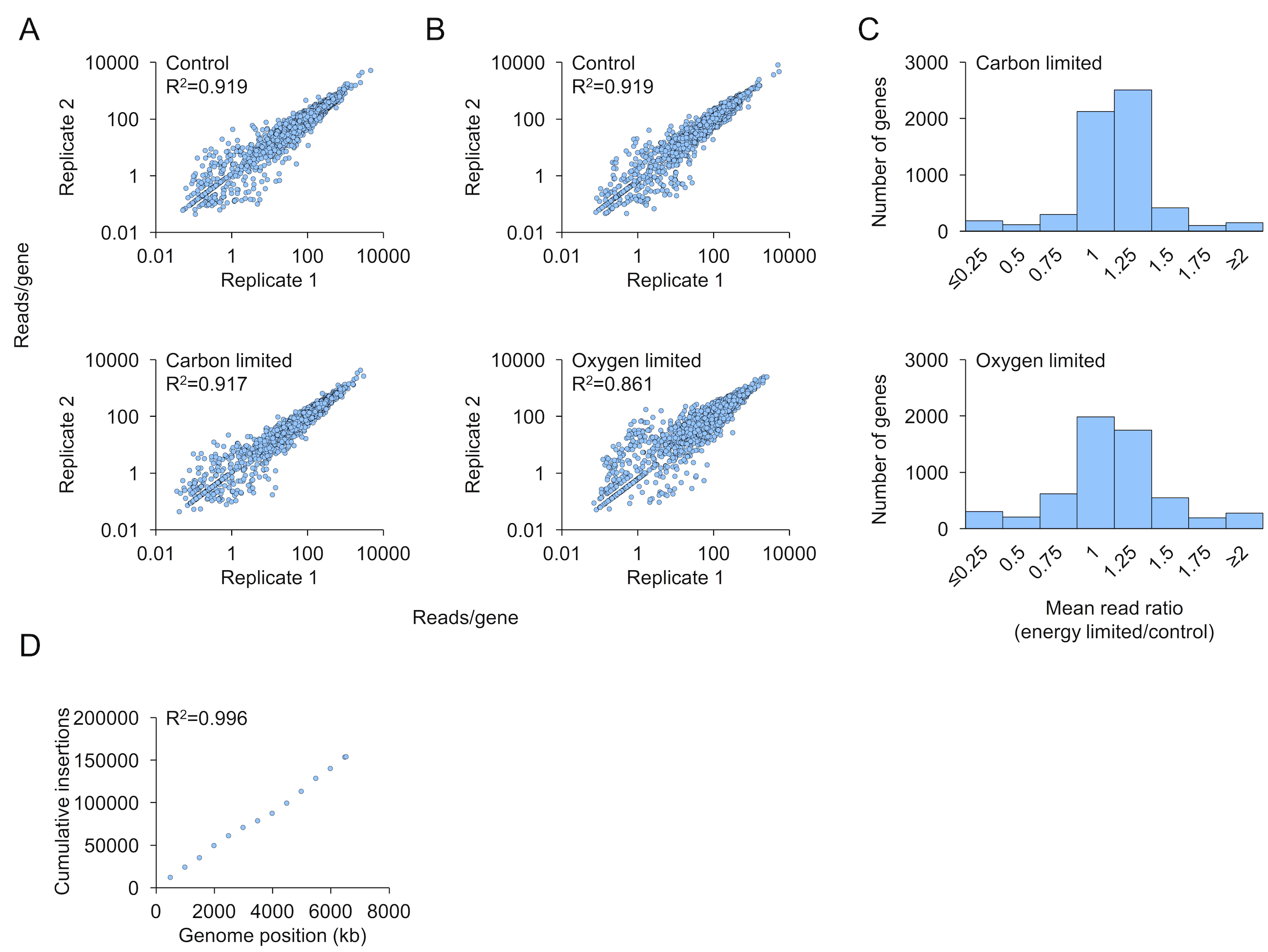

Supplement: FIG S1 [file mbo006173591sf1.tif]

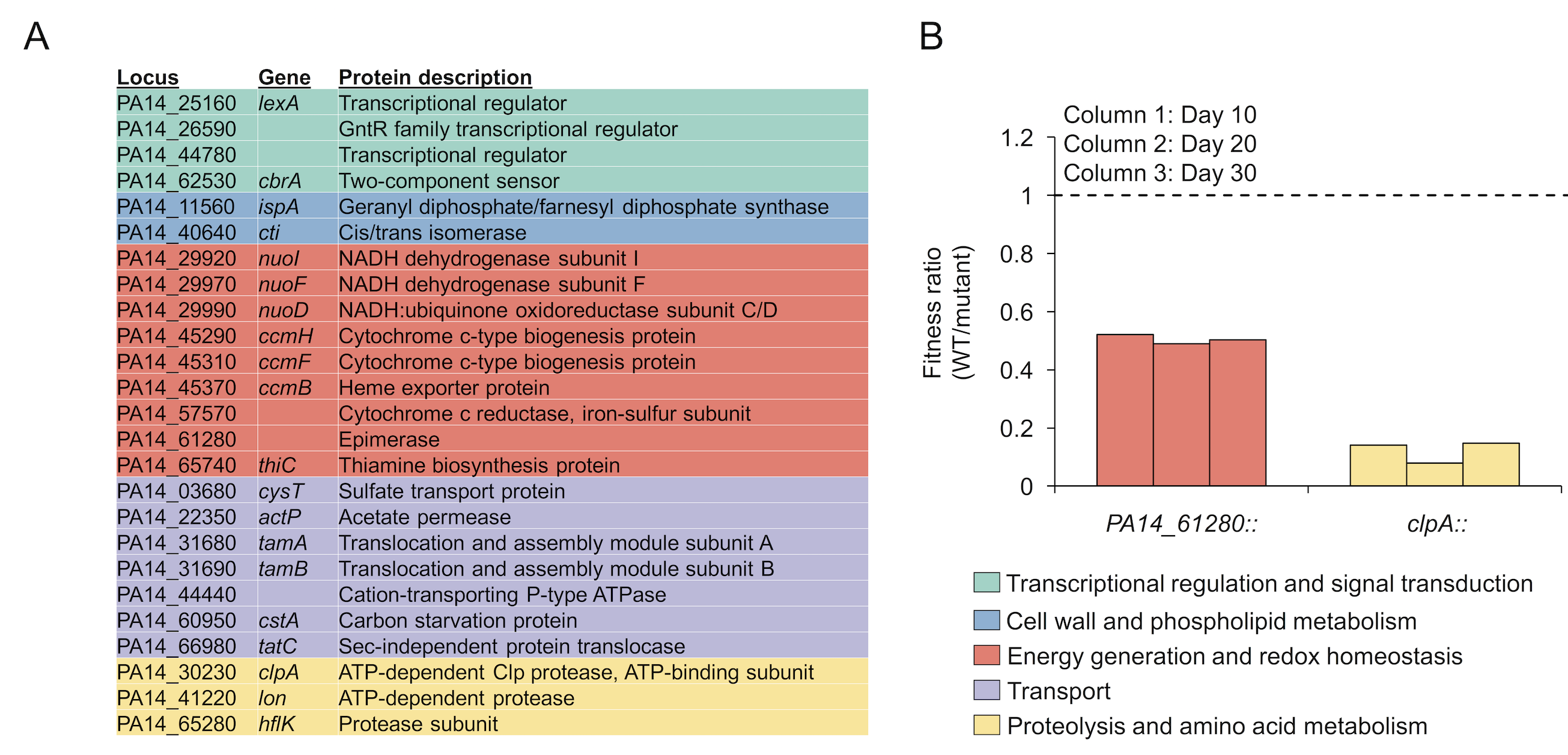

Supplement: FIG S2 [file mbo006173591sf2.tif]
